# Immunization against leukemia inhibitory factor and its receptor suppresses tumor formation of breast cancer initiating cells in BALB/c mouse

**DOI:** 10.1038/s41598-020-68158-0

**Published:** 2020-07-10

**Authors:** Zahra Ghanei, Nahid Mehri, Abbas Jamshidizad, Morteza Daliri Joupari, Mehdi Shamsara

**Affiliations:** Department of Animal Biotechnology, National Institute of Genetic Engineering and Biotechnology, Tehran, Iran

**Keywords:** Breast cancer, Cancer immunotherapy

## Abstract

Immunotherapy is a promising approach for specific targeting of cancer cells. Leukemia inhibitory factor (LIF) regulates several features of cancers and cancer stem cells (CSCs) through binding to LIF receptor (LIFR). In this study, we investigated the consensus of LIF and LIFR immunization on the growth of mouse mammary tumors. For this purpose, mouse LIF and LIFR were designed as truncated proteins, expressed in *E. coli* and then injected to mice as individual and mixed antigens. The results showed the production of neutralizing antibodies and secretion of interferon-γ and interleukin-2 in response to immunization. In continue, the immunized mice were subjected for tumor formation challenge by inoculation of the breast CSCs derived from MC4-L2 cells. Development of the breast tumors was observed in all the control mice, while the tumors appeared in 75% of animals in the LIF group. LIFR injection, individually or in combination with LIF, strongly inhibited the tumor growth to only 25% of the mice. Moreover, a delay in tumor appearance was observed in the immunized mice compared to the controls. Immunostaining of the tumor sections confirmed the expression of LIF and LIFR. In conclusion, LIF and LIFR might be effective targets for immunotherapy of the tumors that express these proteins.

## Introduction

Breast cancer is the most frequently occurring cancer in females. Resistance to therapy and recurrence of disease are two barriers restricting treatment of breast cancer. Novel highly efficient and specific therapeutic approaches causing lower toxicity have to be emerged to overcome these obstacles. Immunotherapy against tumors is an invaluable approach in this regard, which stimulates strong highly specific humoral and cellular immune responses^1–3^.

Leukemia inhibitory factor (LIF) is a pleiotropic protein belonging to the interleukin-6 family cytokines. It is a highly conserved secretory glycoprotein among different species. There is approximately 80% homology between amino acid sequences of human and mouse LIF. This multi-functional cytokine is necessary for maintaining pluripotency state of embryonic stem cells (ESCs) and induced pluripotent stem cells (iPSCs)^4–6^. LIF was identified according to its action on inhibition of mouse myeloid leukemia cells growth and induction of differentiation. LIF also displays several functions on development and progression of cancers. LIF elicits proliferation of different kinds of cancers such as multiple myeloma, colon, prostate and breast cancers^7,8^. LIF upregulation causes tumor resistance to chemotherapy in colorectal cancer. Nasopharyngeal carcinomas with increased level of LIF showed higher rate of growth and radioresistance^9,10^. Wysoczynski et al.^11^ showed that LIF-responsive rhabdomyosarcoma could metastasize to lung several weeks after injection.

LIF exerts its actions through binding to LIF receptor (LIFR) located on cell surface. LIFR is a heterodimeric receptor comprised of LIFRβ and gp130 subunits. LIF first binds to LIFRβ subunit and then to gp130 and makes a functional complex, which can trigger activation of JAK-STAT3 signaling pathway^5^. Overall, LIF and LIFR are expressed in about 80% of human breast cancers^12^.

Cancer stem cells (CSCs), also named as tumor-initiating cells (TICs), are a subset of cancer cells responsible for tumor regeneration, drug resistance and disease recurrence^13^. LIF and LIFR importantly regulate some features of CSCs^14^. Hence, it seems that inhibition of LIF and LIFR might be a therapeutic strategy in suppression of these kind of cells.

In this study, the binding domains of mouse LIF and LIFR were cloned and expressed in *E. coli*. The mouse recombinant truncated LIF (rtLIF) and LIFR (rtLIFR) were purified from bacteria and injected to BALB/c mice. The immunized mice were then subjected for tumor formation challenge by transplantation of breast TICs (BTICs) derived from MC4-L2 cells (thereafter named as MC4-L2^puro^ cells)^15^.

## Results

### Bioinformatic analyses of recombinant proteins

**Figure 1:**
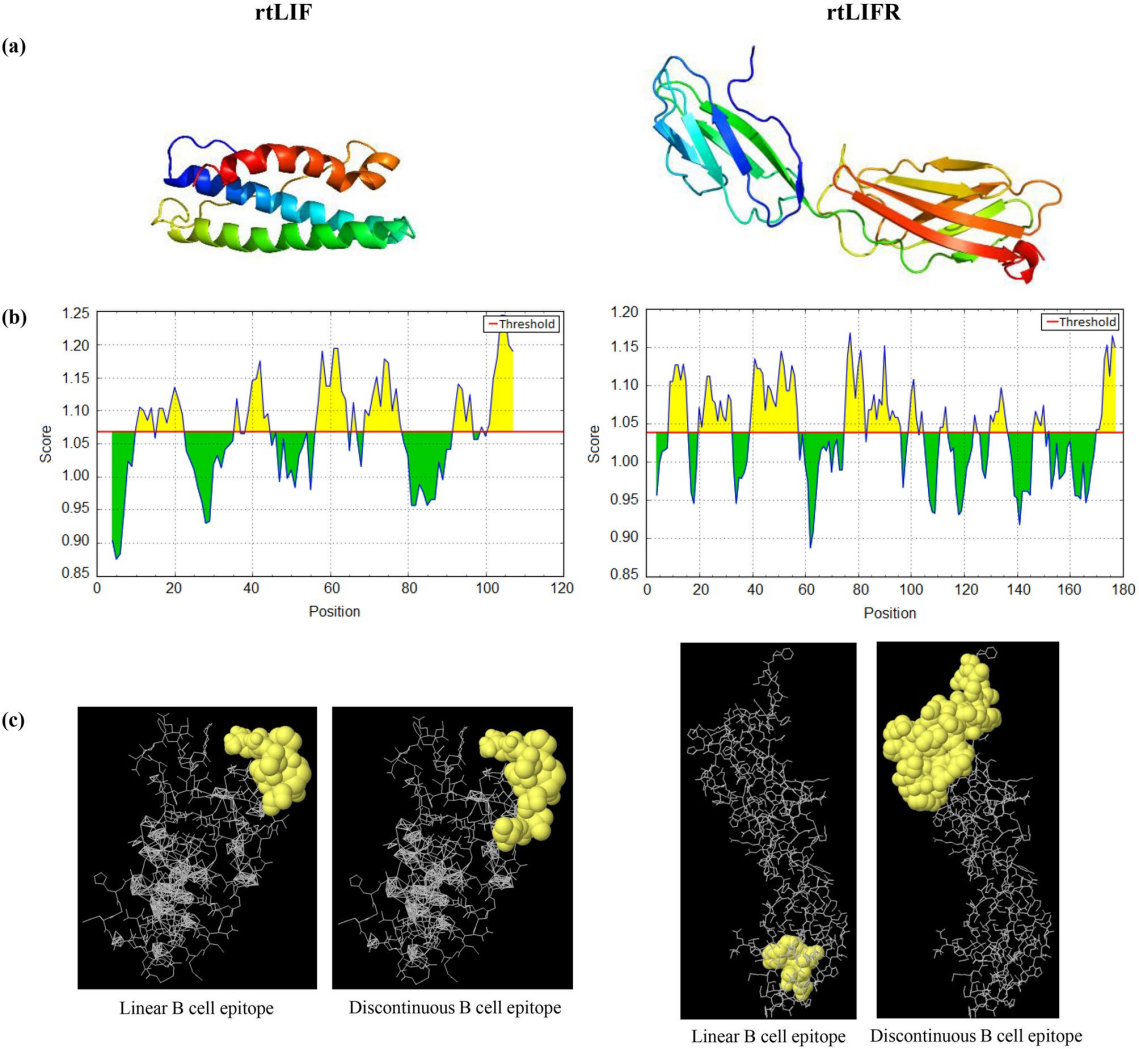
Bioinformatic data of recombinant proteins. (**a**) Prediction of third structure of rtLIF and rtLIFR by Phyre server. (**b**) Kolaskar and Tongaonkar antigenicity prediction tool in IEDB recognized linear B cell epitopes on the rtLIF and rtLIFR. Yellow areas above threshold line were predicted as B cell epitopes. (**c**) Linear and structural B cell epitopes predicted by the Ellipro server.

LIF protein consists of four α-helical bundle topologies (A, B, C, D) with up-up-down-down helix orientation. Two long crossover loops exist between the first two and the last two helices (A–B loop, C–D loop)^16–18^. LIFR possesses an extracellular region with a modular structure containing two cytokine-binding modules, including CBM1 (D1, D2) and CBM2 (D4, D5), separated by an Ig-like domain (D3). These structures are followed by three membrane-proximal fibronectin type-III domains (D6-D8). LIF through its D helix and C-D loop interacts with LIFR at the D3 and D4 domains^19,20^. The rtLIF was expressed as a 110-amino acid fragment of mouse LIF encoding C, D helixes, a part of B helix and C-D loop. The rtLIFR in length of 180 amino acids was a part of mouse LIFR protein bearing D3 and D4 domains (Sequence of both proteins is presented in supplementary data). The stability of both protein transcripts was calculated by the Mfold web server to be -106.68 for rtLIF and-139.39 kcal/mol for rtLIFR. The Phyre predicted third structure of rtLIF protein contained three α-helices plus a loop located between the helices 2 and 3. The rtLIFR structure consisted of two distinct domains with β sheet structures (Fig. 1a).

### Predicted B cell epitopes

Antigenicity of the recombinant proteins was predicted in the Immune Epitope Database (IEDB). Kolaskar and Tongaonkar method in IEBD determined linear (continuous) B cell epitopes (Fig. 1b). Those residues were colored in yellow on the graphs have been computed with much probably to be a part of B cell epitopes. Predicted results for the sequence of rtLIF showed 4 antigenic peptides with a length in range of 6–10 residues. The number of 6 epitopes was also predicted on the rtLIFR sequence in length of 7–19 amino acids.

ElliPro server was used for computing both linear and conformational B cell epitopes. The results showed 4 linear and 5 conformational antigenic peptides on the rtLIF. The number of 9 linear and 4 conformational epitopes was predicted on the rtLIFR (Fig. 1c) (Details of B cell epitopes in supplementary data).

### Predicted T cell epitopes

T cell epitopes were computed by MHC binding tools in IEDB. MHC I binding server predicted the presence of 6 epitopes on the rtLIF. Three MHC I- restricted epitopes were also computed on the rtLIFR. MHC II binding peptide analyses estimated the number of 6 epitopes on the rtLIF, but no one on the rtLIFR (Table 1).

**Table 1:** Prediction of T cell epitopes by IEDB server.

MHC I/II binding peptide	Protein	Allele	Position	Peptide	IC50
**MHC I binding epitopes**					
	rtLIF				
		H-2-Kd	14–27	YRMVAYLSASLTNI	29.09
		H-2-Kd	15–28	RMVAYLSASLTNIT	51.71
		H-2-Kd	18–31	AYLSASLTNITRDQ	63.15
		H-2-Kd	16–29	MVAYLSASLTNITR	65.22
		H-2-Kd	17–30	VAYLSASLTNITRD	68.55
		H-2-Db	21–34	SASLTNITRDQKVL	75.67
	rtLIFR				
		H-2-Kk	124–137	FESISGKSAVFHRI	9.23
		H-2-Kk	120–133	EYTLFESISGKSAV	56.77
		H-2-Kk	56–69	IPVSENSGTNIIFI	74.82
**MHC II binding epitopes**					
	rtLIF				
		H2-IAd	11–25	VELYRMVAYLSASLT	30.5
		H2-IAd	12–26	ELYRMVAYLSASLTN	31.4
		H2-IAd	13–27	LYRMVAYLSASLTNI	38.5
		H2-IAd	10–24	LVELYRMVAYLSASL	38.7
		H2-IAd	14–28	YRMVAYLSASLTNIT	60.8
		H2-IAd	9–23	KLVELYRMVAYLSAS	75.6

### Expression and purification of recombinant proteins

**Figure 2:**
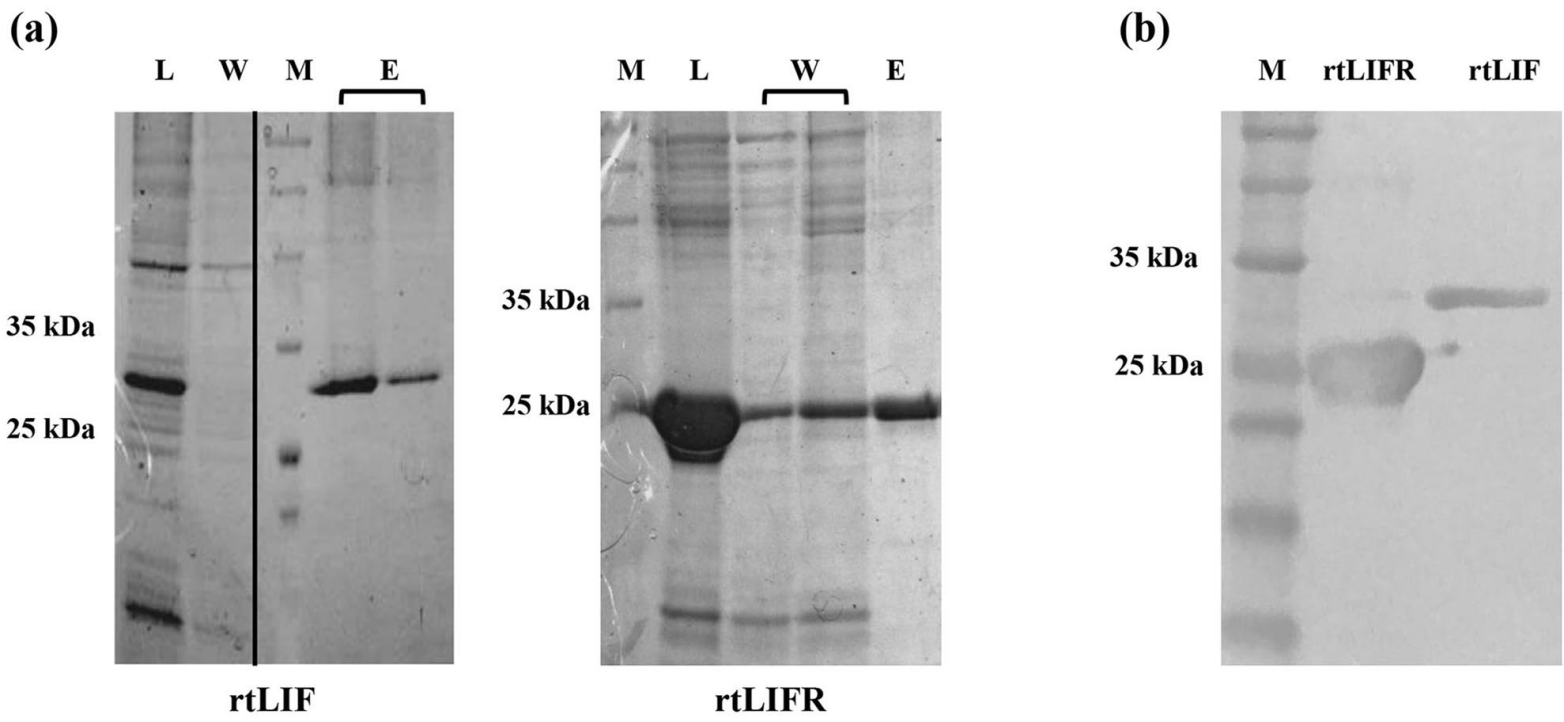
Expression of rtLIF and rtLIFR proteins in *E. coli*. (**a**) SDS-PAGE of induced bacteria lysates followed by Ni-column purification showed the rtLIF and rtLIFR protein bands with the molecular weights of 30 and 25 kDa, respectively. M: protein size marker; L: bacterial lysate; W: wash; E: elution. (**b**) Western blotting using histidine-tag antibody confirmed expression of both proteins. Full-length of gels and blot are presented in supplementary data.

*E. coli* codon-optimized rtLIF and rtLIFR sequences were chemically synthesized and received in pUC58 plasmids. The protein sequences were fused to a tetanus-derived peptide for improvement of immune stimulation and histidine tag residues for Ni–NTA purification. For protein expression, the rtLIFR gene was inserted into a pET21b plasmid and the rtLIF was subcloned into a pET30-KSI vector. The optimum condition of protein expression was determined in 37 °C and 0.1 mM IPTG. SDS-PAGE of bacterial lysates revealed protein bands in the size of 30 and 25 kDa according to the molecular weight of rtLIF and rtLIFR proteins, respectively (Fig. 2a lane L). Both proteins were insoluble in *E. coli* and purified under denaturing condition using the Ni-columns (Fig. 2a lane E). The proteins were confirmed in western blot using a histidine tag recognizing antibody (Fig. 2b).

### Raising up neutralizing antibodies

**Figure 3:**
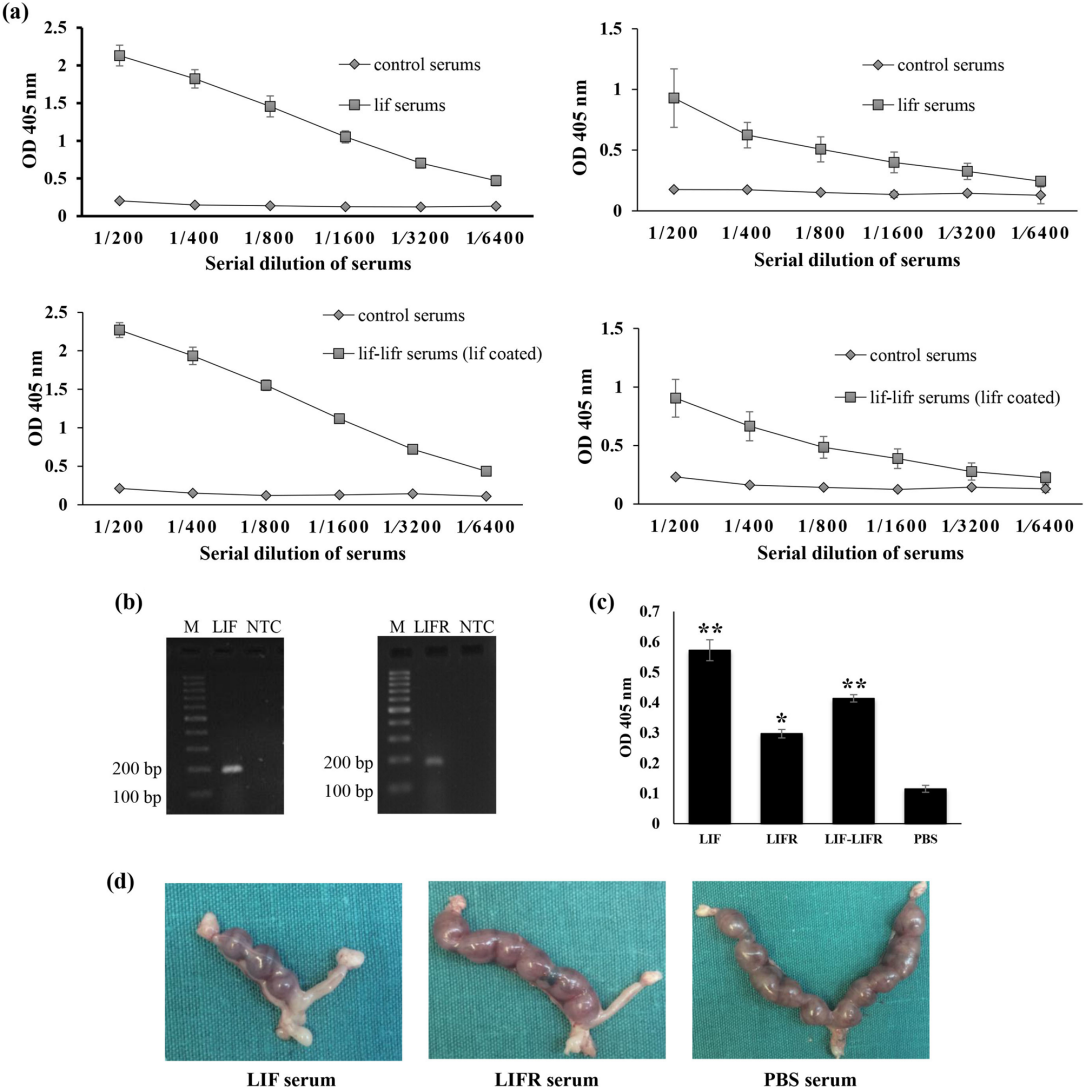
Titration and functional assay of LIF and LIFR antibodies. (**a**) Serially diluted antiserums recognized the rtLIF and rtLIFR proteins coated in ELISA plates. (**b**) RT-PCR revealed the expression of both LIF (184 bp) and LIFR (167 bp) by the MC4-L2^puro^ cells (**c**) LIF and LIFR serums reacted with LIF and LIFR proteins derived from MC4-L2^puro^ cells. Data represent mean ± SD of three independent experiments, * *p* < 0.01, **p < 0.001. (**d**) Neutralization assay showed intrauterine injection of LIF or LIFR antiserums in one horn blocked uterus implantation of embryos. The other horn was left noninjected for pregnancy control.

Antibody production in response to protein injection was evaluated by ELISA. The titer of antibodies using serially diluted serums was 1:6,400 for LIF and 1:3,200 for LIFR (Fig. 3a). To study interaction of antibodies with cell-derived LIF and LIFR proteins, reverse transcription-PCR showed expression of both genes by the MC4-L2^puro^ cells (Fig. 3b). Total protein of MC4-L2^puro^ cells was used in ELISA and the results showed that the produced antibodies could interact with LIF and LIFR proteins from cell resources (Fig. 3c).

Neutralization activity of the secreting antibodies was studied on the fate of embryo implantation. LIF and LIFR are both essential for embryos implantation into uterine wall. Therefore, for neutralization assay, the antiserums were injected into uterine cavity of pregnant mice at one horn and the other horn was left noninjected for pregnancy control. The results showed that blockage of embryo implantation at the horn received LIF and LIFR antiserums, but not at the horn received control serum (Fig. 3d).

### Enhancement of IFN-γ and IL-2

**Figure 4:**
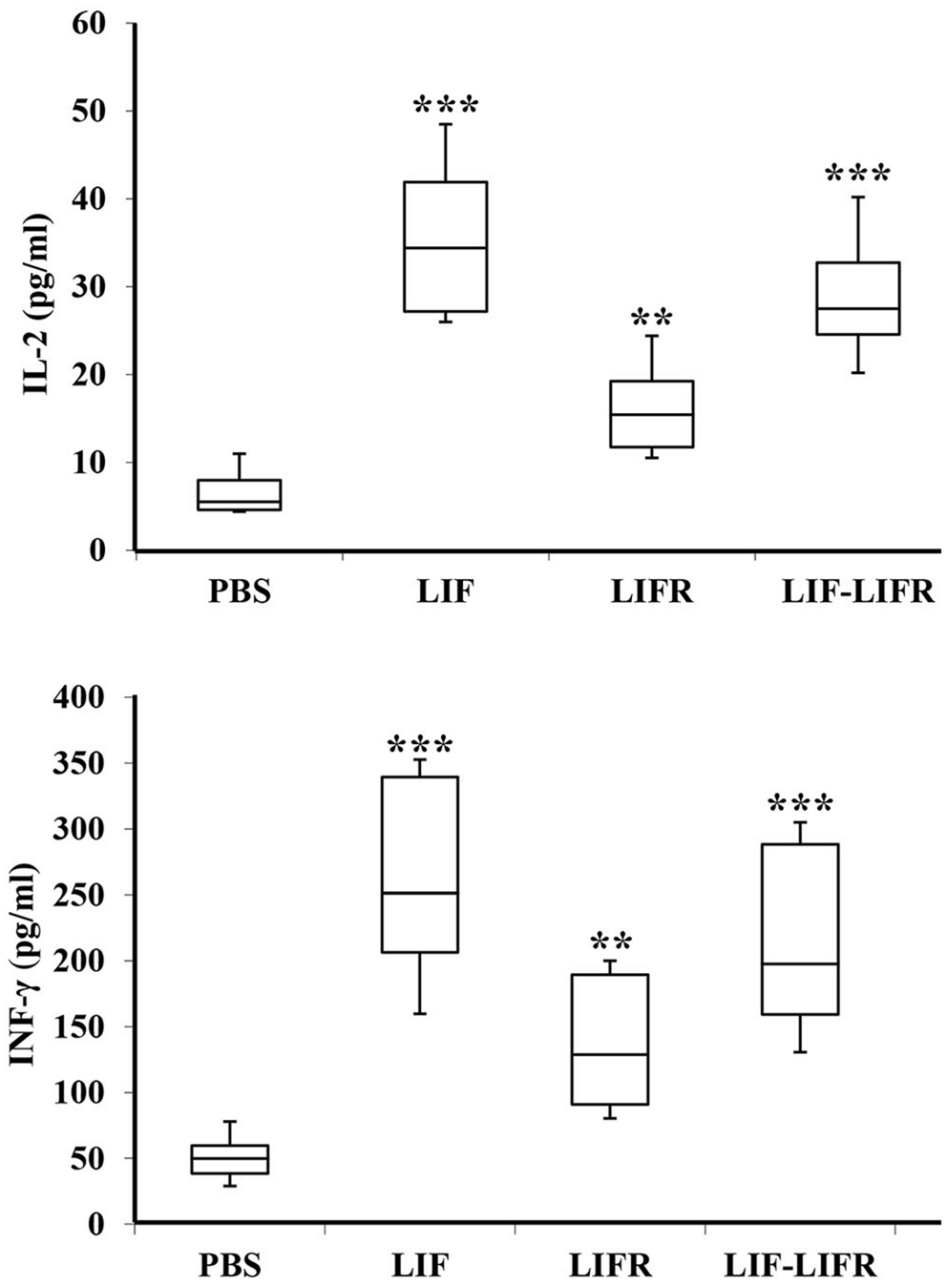
Cytokine assay in the immunized serums. Serum IFN-γ and IL-2 increased in response to immunization of mice with the recombinant proteins. Data represent mean ± SD of three independent experiments, **p < 0.001, ***p < 0.0001.

Level of IFN-γ and IL-2 cytokines were measured in serum samples. These cytokines are secreted in response to activation of cellular immunity. Cytokine assay revealed a significant enhancement of IFN-γ and IL-2 (Fig. 4). The level of INF- γ was raised up from 49.75 pg/ml in control serum to 251.25 and 128.6 pg/ml in LIF and LIFR antiserums, respectively. IL-2 amount was also increased to 34.4 and 15.45 pg/ml in the animals immunized with rtLIF and rtLIFR, respectively. Enhancement of IFN-γ and IL-2 was also measured in the group received combined antigens.

### Tumor formation decrease due to immunization

**Figure 5:**
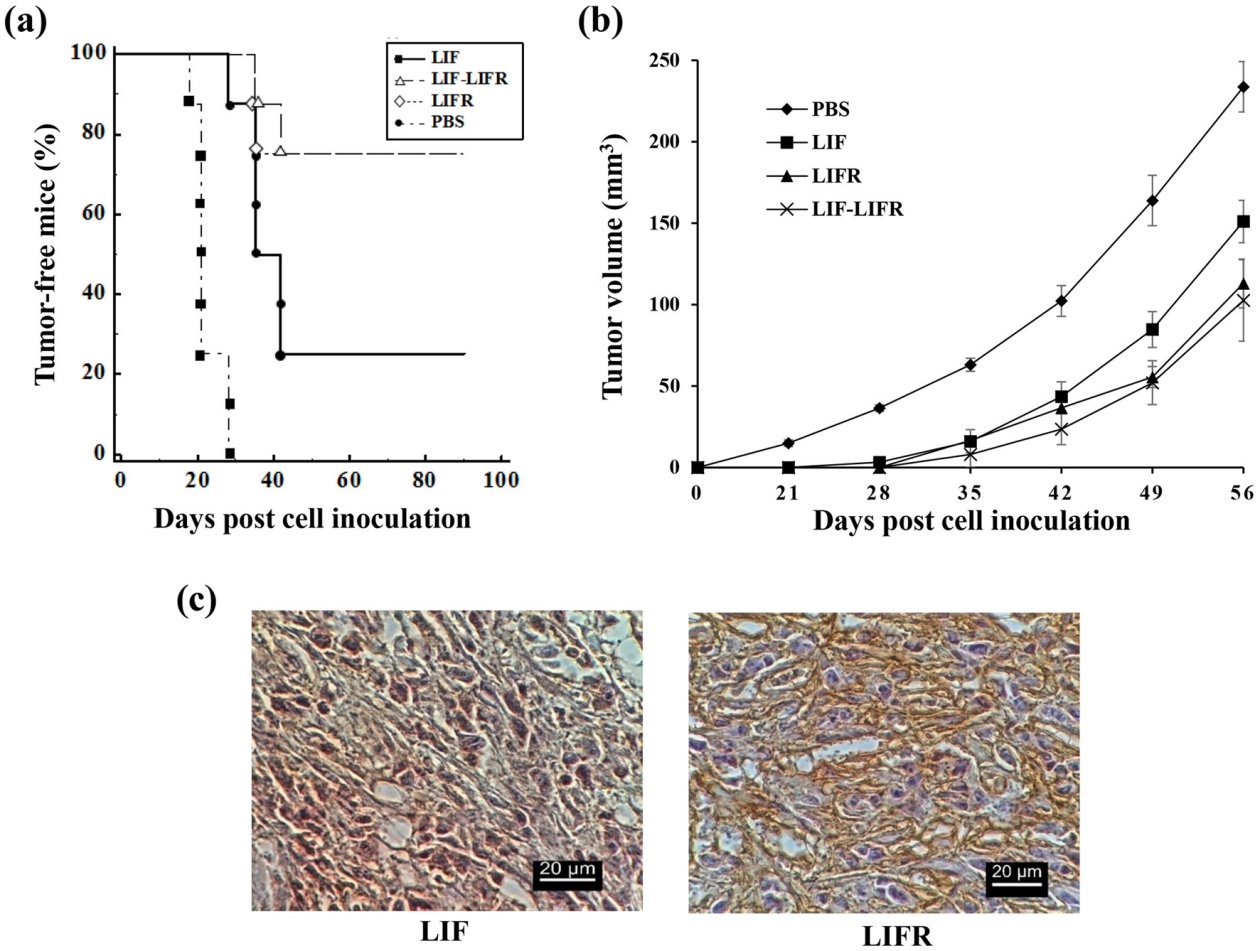
Immunoprotective effects of rtLIF and rtLIFR on tumor formation. (**a**) Kaplan–Meier survival analysis showed development of breast tumor by over the time. A significant increase in the number of tumor free mice was observed in the test groups compared to the control one (*p* < 0.002, log-rank test) (**b**) Comparison of tumor volume between the experimental groups. Statistical analyses by two-way ANOVA showed a significant decline in tumor volume of the immunized animals compared to the controls (*p* < 0.0005) (**c**) IHC staining on the MC4-L2^puro^-derived tumors showed the expression of both LIF and LIFR.

Tumor formation challenge in the immunized mice was performed by injection of MC4-L2^puro^ cells into breast fat-pad. The mice were monitored up to three months for tumors. The tumors were grown to palpable size in all control mice four weeks after cell inoculation. However, the number of tumors in the LIF group was restricted to 6 mice out of 8 (75%). The highest decline was observed in the LIFR and LIF/LIFR groups, in which only 25% of mice (2 out of 8) developed tumors (Fig. 5a). The rest of immunized mice lived tumor-free. Furthermore, delay in the appearance of tumors was observed in the immunized mice compared to the controls (Fig. 5b).

Expression of LIF and LIFR by the breast tumors were studied. The tumors were dissected and subjected for IHC using specific antibodies. Immunostaining results showed expression of both LIF and LIFR by the tumors (Fig. 5c).

## Discussion

Metastasis, drug resistance, and recurrence are critical obstacles in the treatment of breast cancer, which are mainly mediated by CSCs^21,22^. There are many interests in the identification of new therapeutic approaches, which can target and eliminate this subset of cancer cell population^23^. However, one of the challenging issues in this regard is the high heterogeneity among CSCs and cancer cells, which makes it difficult to identify a common targeting molecule on cancer cells^24^. Cancer immunotherapies using monoclonal antibodies, immune provoking cytokines, engineered cells, and a combination of them are promising approaches that stimulate the defense system to fight cancer cells and prevent relapses^25^. Hence, in this study, we investigated the consensus of LIF and LIFR immunization on the development of breast tumors in mice.

LIF:LIFR signaling activates a group of intracellular pathways, which mediate progression, migration, and invasion of tumors^9,12^. The function of LIF:LIFR signaling in stemness and progression of cancers has been studied previously. For instance, siRNA-mediated suppression of LIF restricted proliferation, attachment, migration, and colony formation of human melanoma cells^26^. Similarly, LIFR inhibition suppressed metastasis of pancreatic carcinoma and rhabdomyosarcoma, reduced stemness and growth of ovarian and breast cancers, and promoted apoptosis^11,27^. All this evidence indicates that LIF:LIFR axis might be an attractive target for cancer immunotherapy.

In this study, we investigated the consensus of LIF and LIFR immunization on the growth of breast tumors after transplantation of the BTICs into breast fat-pad. Truncated mouse LIF and LIFR proteins were designed, characterized bioinformatically, and expressed in a prokaryotic system. The proteins were purified and injected into the mice, and the immune system stimulation was evaluated by the assessment of humoral and cellular immunities. The results indicated the secretion of antibodies due to injections. Antibody titration assay showed higher production of LIF antibodies compared with LIFR antibodies. IFN-γ and IL-2 assessments showed the induction of cellular immunity after injections. More cytokines were secreted in the LIF-injected mice than in the LIFR-injected mice. These results indicated that rtLIF might display stronger immunogen properties than rtLIFR. It was also consistent with our bioinformatic findings, in which a greater number of conformational epitopes and MHC II binding peptides were predicted on the rtLIF.

LIF is frequently upregulated in different types of human tumors, including nasopharyngeal carcinoma, melanoma, breast, colorectal and lung cancers^9,10,26,28^. LIF mediates several functions in tumor development and progression. In contrast to inhibition of leukemia cells growth, LIF often promotes the development and progression of many solid tumors. Binding of LIF to LIFR activates multiple signaling pathways, including STAT3, AKT, and mTOR^9^. Overexpression of LIF increased migration and invasion rate of tumor cells in both in vitro and in vivo^11,28^. Yue and colleagues reported that LIF provoked epithelial to mesenchymal transition (EMT) in breast cancer cell lines via the induction of miR-21^29^. Furthermore, LIF promoted tumor progression of estrogen receptor (ER) positive breast cancer cells, such as MCF-7 and T47-D, in a dose-dependent fashion^7^. MC4-L2 cells have been derived from an ER-positive breast tumor^30^. We previously derived the MC4-L2^puro^ cells from a MC4-L2 population and characterized them as CSC-like cells^15^. Inoculation of these cells to BALB/c mice induced breast tumors. Since LIF gene expression was confirmed in these cells and derived breast tumors, we studied the effect of LIF immunization on tumor development. The results showed that the tumor appearance was restricted to 75% of the immunized mice, and the remaining animals lived tumor-free until the end of the experiments.

LIFR plays both like an oncogene and like a suppressor gene in cancers. LIFR suppressed the metastasis of hepatocellular carcinoma via the downregulation of PI3K/AKT^31^. However, it displayed a pro-metastatic function in melanoma and stimulated cell migration by STAT3-mediated pathway^32^. Both functions of LIFR have been recognized in breast cancer. Chen and co-workers reported that LIFR inhibited metastasis of breast tumor through the inactivation of YAP protein in Hippo-YAP pathway^33^. In addition, LIFR inhibited cell migration and invasion through activation of STAT3 signal^34^. A recent study showed that LIFR upregulation suppressed metastasis and induced dormancy of disseminated breast cancer cells in bone marrow^35^. On the other hand, the oncogenic role of LIFR has been known in triple-negative breast cancer MBA-MD-231 cells. It is probably because of that all the effects of LIF:LIFR signaling in these cells are conducted by AKT/mTOR pathway instead of STAT3^28^. Dhingra and others showed that exogenous LIF stimulated the growth of ER-positive breast cancer cells in cultured cells through binding to LIFR^12^. Furthermore, ILEI is an oncogenic cytokine that can bind to LIFR and trigger EMT and CSC traits of breast tumors via STAT3 activation^36^. Viswanadhapalli and co-workers discovered a new small molecule, which could inhibit the oncogenic action of LIFR in MBA-MD-231 cells^37^. These findings might introduce LIFR as an immunotherapy target in ER-positive breast tumors and triple-negative breast cancers. In this study, we showed that tumor formation in the LIFR immunized group happened in only 25% of mice. The same results were also achieved in the mice immunized by LIFR combined with LIF.

We found an inverse relationship between the rate of tumor formation and the defense responses induced by the injection of LIF and LIFR. Although the LIF immunization caused higher production of antibodies and cytokines, more animals lived tumor-free in the LIFR-received groups. This might be described by the fact that in addition to LIF, four other cytokines, including oncostatin M, cardiotrophin-1, ciliary neurotrophic growth factor, and cardiotrophin-like cytokine also bind to LIFR and trigger downstream signals^5^. Therefore, these overlapping cytokines might compensate for the loss of LIF function, while blockage of LIFR inhibits the action of LIF and overlapping cytokines. This might be a reason for the finding that LIFR inhibition is a more efficient approach to prevent tumor formation than LIF inhibition.

Neutralizing activity of the antiserums on LIF and LIFR proteins was evaluated by blocking the uterine implantation of embryos. LIF and LIFR are both essential for embryonic stage development and uterine implantation of blastocysts. According to a prior study, transgenic mice encoding a non-functional LIF completely failed in the implantation stage of embryos^38^. Moreover, the administration of a LIF antagonist blocked the embryo implantation in mouse^39^. Having considered these studies, the neutralizing activity of the antiserums was tested by the uterine injection of LIF and LIFR. The results showed that intrauterine injection of the immunized serums at one horn completely inhibited the embryo implantation, while in the non-injected horn, the embryos were implanted successfully. This finding indicated that the released antibodies could neutralize the function of endogenous LIF and LIFR proteins.

Cytokines regulate the activity of innate and adaptive immune systems and enable defensive cells to fight infections and malignant cells. IFN- γ, and IL-2 are cytokines with known antitumor effects. In our study, the measurement of these cytokines revealed the enhancement of both after immunization. IFN-γ plays importantly in the provocation of immune responses. It can mediate macrophage activation and induction of MHC I/II and co-stimulatory molecules on antigen-presenting cells^40,41^. IL-2 is another regulatory cytokine, which is produced predominately by antigen-simulated CD4^+^ T cells. It is also secreted by other immune cells, like CD8^+^ T cells, natural killer cells, and activated dendritic cells. IL-2 regulates activation and expansion of those immune cells that participate in tumor regression^42,43^.

## Conclusion

In this study, we investigated the antitumor effect of immunization against LIF and LIFR. Our findings indicated that immune-mediated blockage of LIF and LIFR delayed or event prevented tumor growth. Moreover, immunotherapy of LIFR was a more efficient approach in the inhibition of tumor growth. In conclusion, according to the findings of this study LIF and LIFR can be considered in cancer targeted therapy, especially to fight with those tumors that express these proteins such as tumor-initiating cells.

## Materials and methods

### Cell culture

The MC4-L2^puro^ cells were cultured in DMEM/F12 medium supplemented with 1% knockout serum replacement (KSR) (Gibco), 2 ng/ml LIF (Millipore), 10 ng/mL recombinant epidermal growth factor (rEGF) (Royanbitech, RP-1102), 10 ng/mL basic fibroblast growth factor (bFGF) (Royanbitech, RP-1101) and 0.5% bovine serum albumin (BSA) (Bio Basic Inc, AD0023).

### RNA extraction and RT-PCR

To evaluate the expression of LIF and LIFR by the MC4-L2^puro^ cells^15^, total RNA was extracted using Tripure Isolation Reagent (Roche) according to manufacturer’s protocol. Reverse transcription-PCR (RT-PCR) was performed using the sets of primers including, mLIF-F: 5′-CTGCTGGTTCTGCACTGGAAAC-3′, R: 5′-GCTCCCCTTGAGCTGTGTAATAG-3′, mLIFR-F: 5′-CGGAAGCGAGAATGGATTAAGGA-3′, R: 5′-GACCGAGATTCCAGGACTTCAAC-3'.

### Selection of immunogenic domains in LIF-LIFR binding site

Sequences of LIF and LIFR binding sites were selected by literature review^16–20^. Truncated forms of mouse LIF and LIFR, which respectively nominated as rtLIF and rtLIFR, were designed and subjected for in silico analyses. RNA stability of both proteins was calculated by Mfold online tool (https://unafold.rna.albany.edu/?q=mfold). ProtParam server (https://web.expasy.org/protparam) computed molecular weight of the proteins. Third structure of the proteins was predicted according to the proteins amino acids using the Phyre server (https://www.sbg.bio.ic.ac.uk/phyre2/html/page.cgi?id=index).

### Prediction of B cell and T cell epitopes

Antigenicity and epitope mapping of the recombinant proteins were predicted using the tools available in the Immune Epitope Database (IEDB) server (https://tools.immuneepitope.org). Kolaskar and Tongaonkar antigenicity methods predicted the probability of linear specific regions in proteins, which can bind to B cell receptor. Application of this method can predict antigenic determinants with about 75% accuracy^44^. ElliPro was another tool in IEDB, which was used for prediction both linear and conformational B cell epitopes on the proteins (https://tools.immuneepitope.org/tools/ElliPro/iedb_input). ElliPro predicts epitopes from sequence and also 3D structures of proteins in PDB format based upon solvent-accessibility and flexibility^45^. T cells epitopes were also computed using the MHC I and MHC II binding peptides prediction tools available in IEDB. T cell epitopes were predicted using IEDB recommended method and epitopes with IC50 < 100 nm were selected.

### Gene synthesis, protein expression and purification

The *E. coli* codon-optimized sequences encoding mouse truncated forms of LIF (rtLIF) and LIFR (rtLIFR) were chemically synthesized and received in pUC58 vectors. Both proteins were fused to a tetanus-derived peptide (QYIKANSKFIGITEL) for enhancement of protein immunogenicity. For protein expression, the synthetic rtLIF was subcloned into a pET30-KSI plasmid using the *Nco*I and *Xho*I enzymes in frame with a Ketosteroid isomerase (KSI) sequence at N-termianl and a histidine tag at C-terminal. KSI has been previously inserted to a pET30a plasmid to increase protein stability. The rtLIFR fragment was inserted into a pET21b plasmid at the sites of *Nde*I and *Xho*I enzymes to replace the pelB signal peptide. The proteins were expressed in *E. coli* strain BL21 (DE3) by adding isopropyl-b-D-thiogalactopyranoside (IPTG) as inducer. Bacterial cell lysates were analyzed on 12% SDS-PAGE and then the proteins were purified under a denaturing condition by means of a nickel affinity chromatography (Qiagen) as the manufacturer's recommendation. The purified proteins were dialyzed, lyophilized and stored at − 70 °C until use. Prior to immunization, the powders were dissolved in water and the protein contents were quantified using the Bradford method^46^.

### Western blotting

Precipitates were separated on 12% SDS‐PAGE and blotted onto a PVDF membrane. Blocking was carried out in 40 mM Na_2_HPO_4_, 7 mM NaH_2_PO4, 1% milk powder, 0.05% w/v sodium azide, 0.5% w/v Tween‐20, and pH 7.5. The membrane was incubated with a histidine tag antibody (Abcam) at dilution of 1:5,000. Bound antibodies were detected by a goat anti‐mouse antibody conjugated to horseradish peroxidase (HRP) (diluted 1:10,000; Invitrogen). The protein bands were appeared by adding 3, 3′ diaminobenzidine tetrahydrochloride (DAB) (Sigma).

### Immunohistochemistry

Tumors were fixed in 10% formalin and blocks were prepared by embedding tumors in paraffin. Blocks were sectioned at 5 µm thickness. Sections were then de-waxed and rehydrated and endogenous peroxidases were deactivated with hydrogen peroxide. Sections were then boiled in TBS buffer and blocked in 5% serum for 1 h. Primary antibodies were incubated overnight at 4 °C at 1:100 for LIFR (Abcam) and LIF (LSBio). HRP anti-rabbit secondary antibodies (diluted 1:2000; Invitrogen) were incubated for 1 h at room temperature and the slides were washed for 1 h in PBS. Bound antibodies were visualized by incubation with 3,3′ diamino-benzidine tetrahydrochloride (DAB, DAKO). Finally, slides were rinsed in tap water, counter-stained with hematoxylin and mounted under cover slide.

### Mouse immunization

This study was conducted in accordance with all protocols approved by the National Institute of Genetic Engineering and Biotechnology Animal Care Committee. Female BALB/c mice at age of 5–6 weeks old were ordered from the Royan Institute. The mice fed with standard diet and kept in a room with controlled temperature (22 ± 2 °C) and humidity under a 12 h light–dark cycle for two weeks before starting immunization. Preimmune serums were prepared before starting the immunization. Thirty-two females were divided into four experimental groups, including three test and one control groups. Test mice were immunized against LIF, LIFR and both of them, while controls received PBS. Antigens were applied subcutaneously in the amount of 35 μg antigen was injected to each mouse in the LIF and LIFR groups. Animals in the combination group received 70 μg of mixed antigens. The first injection was administered by mixing the antigens with equal volume of complete Freunde’s adjuvant. In addition, three boosters with incomplete Freunde’s adjuvant were received by the mice in weeks 4, 6 and 8.

### Immunization assay

Mice were bled via facial vein after receiving the last booster. Serums were tested for production of LIF- and LIFR-specific antibodies in an ELISA. Briefly, 96-well plates were coated at 4 °C overnight with 200 ng/well of rtLIF and rtLIFR in a coating buffer (100 mM Na_2_CO_3_, 50 mM NaHCO_3_ and pH 9.6). The plates were incubated with 1:200, 1:400, 1:800, 1:1,600, 1:3,200, 1:6,400 dilutions of the immune serums in TEN-TC buffer (50 mM Tris, 12 mM EDTA, 1.5 M NaCl, 0.5% Tween 20 and 2% casein) for 1 h at 37 °C. Washing with TEN-T buffer (TEN-TC without casein) was followed by incubation with polyclonal goat anti-mouse-IgG HRP-conjugated antibody (Invitrogen) diluted at ratio of 1:1,000 in TEN-TC buffer for 1 h at 37 °C. ABTS (Roche) and H_2_O_2_ was added and incubated for 15 min in darkness. In the end, the absorbance was measured at 405 nm by an ELISA instrument.

### Neutralization assay of antiserums

LIF and LIFR are both essential for uterine implantation of embryos and their blockage prevent pregnancy. Regarding this, we applied the LIF and LIFR antiserums to evaluate neutralization. Nine females at the age of 8-week old were divided into three groups and paired with males. Plaque-positive mice were separated and kept in separate cages. At day 3.5 dpc (days post coitum), females were anesthetized using intraperitoneal injection of ketamine and xylazine and the mouse's side was opened with surgery. One of the uterine horns was fixed outside by a clamp. Each serum, including two immunized serums and one PBS serum, was diluted at ratio of 1:50 in PBS and separately injected into the fixed horn. The other horn was left noninjected in order to pregnancy control. Females were sacrificed at day 12.5 dpc and dissected and embryo implantation was observed in horns.

### Tumor formation challenge

Tumor formation challenge was investigated by injection of the MC4-L2^puro^ cells as BTICs. The mice were injected at inguinal fat pad with 10^6^ MC4-L2^puro^ cells 5 days after the last immunization. Tumor growth was monitored two times a week using Vernier calipers in two dimensions: A (long diameter) and B (short diameter). Tumor volume was calculated with the formula: V = (A × B2)/2, and expressed in mm^3^. Mice were followed for 3 months for emerging tumors. At last, the mice were dissected and carefully examined for formation of tumor.

### Measurement of IFN-γ and IL-2

Serums were harvested from the immunized mice and the content of interferon-γ (IFN-γ) and interleukin-2 (IL-2) were measured in by commercially available ELISA kits (Sigma) according to the manufacture’s protocol.

### Statistical analysis

The significance of quantitative data was statistically analyzed by t-test and two-way analysis of variance (ANOVA). Survival curves were compared using the log-rank test. The values were considered statistically significant when *p* < 0.05.

## Supplementary information

Supplementary Information.
