# Supplementary material for: Immunization against leukemia inhibitory factor and its receptor suppresses tumor formation of breast cancer initiating cells in BALB/c mouse
Source: Sci Rep. 2020 Jul 10;10:11465. doi: 10.1038/s41598-020-68158-0 (PMC7351713; doi:10.1038/s41598-020-68158-0)

Immunization against Leukemia Inhibitory Factor and Its Receptor Suppresses Tumor Formation of Breast Cancer Initiating Cells in BALB/c Mouse

Zahra Ghanei[1], Nahid Mehri[1], Abbas Jamshidizad[1], Morteza Daliri Joupari[1], Mehdi Shamsara[1,2*]

[1]Department of Animal Biotechnology, National Institute of Genetic Engineering and Biotechnology, Tehran, Iran

[2]Pediatric Cell Therapy Research Center, Tehran University of Medical Sciences, Iran

*Corresponding author: Dr. Mehdi Shamsara, E-mail: shamsa@nigeb.ac.ir, Tel: +982144787414, Fax: +982144787399

Supplementary data

rtLIF protein sequence:

Tetanus peptide | Part of B helix

PWQYIKANSKFIGITELAAAHGNGTEKTKLVELYRMVA

C helix | C-D Loop

YLSASLTNITRDQKVLNPTAVSLQVKLNATIDVMRGLLS

D helix

NVLCRLCNKYRVGHVDVPPVPDHSDKEAFQRKKLGCQ

His tag

LLGTYKQVISVVVQAFAAALEHHHHHH

rtLIFR protein sequence:

Tetanus peptide

MQYIKANSKFIGITELAAANTETNVFPQDKVVLAGSNMTICC

Ig-like domain (D3)

MSPTKVLSGQIGNTLRPLIHLYGQTVAIHILNIPVSENSGTNIIF

ITDDDVYGTVVFAGYPPDVPQKLSCETHDLKEIICSWNPGRI

Part of CBM2 (D4)

TGLVGPRNTEYTLFESISGKSAVFHRIEGLTNETYRLGVQMH

His tag

PGQEIHNFTLTGRNPLGQAQSAVVINVTAAALEHHHHHH

Supplementary Table 1: Kolaskar and Tongaonkar method predicted peptides

No.	Start	End	Peptide	Length
**LIF**				
1	16	22	MVAYLSA	7
2	39	44	VSLQVK	6
3	57	64	SNVLCRLC	8
4	69	78	VGHVDVPPVP	10
**LIFR**				
1	9	15	QDKVVLA	7
2	20	32	TICCMSPTKVLSG	13
3	39	57	RPLIHLYGQTVAIHILNIP	19
4	75	82	YGTVVFAG	8
5	84	96	PPDVPQKLSCETH	13
6	130	136	KSAVFHR	7

Supplementary Table 2: ElliPro predicted linear epitope(s)

No.	Start	End	Peptide	Number of residues	Score
**LIF**					
1	50	58	KVLNPTAVS	9	0.808
2	18	24	SHGNGTE	7	0.707
3	79	107	CRLCNKYRVGHVDVPPVPDH SDKEAFQRK	29	0.701
4	122	128	SVVVQAF	7	0.564
**LIFR**					
1	70	76	ITDDDVY	7	0.829
2	151	160	QMHPGQEIHN	10	0.786
3	125	132	FESISGKS	8	0.76
4	173	192	QSAVVINVTGSQYIKANSKF	20	0.737
5	3	7	TETNV	5	0.716
6	24	57	CMSPTKVLSGQIGNTLRPLIH LYGQTVAIHILNI	34	0.683
7	96	101	THDLKE	6	0.657
8	140	144	GLTNE	5	0.581
9	16	19	AGSN	4	0.533

Supplementary Table 3: ElliPro predicted discontinuous epitope(s)

No.	Residues	Number of residues	Score
**LIF**			
1	_:K50, _:V51, _:L52, _:N53, _:P54, _:T55, _:A56, _:V57, _:S58, _:V61, _:K62	11	0.775
2	_:C79, _:R80, _:L81, _:C82, _:N83, _:K84, _:Y85, _:R86, _:V87, _:G88, _:H89, _:V90, _:D91	13	0.712
3	_:P96, _:D97, _:H98, _:S99, _:D100, _:K101, _:E102, _:A103, _:F104, _:Q105, _:R106, _:K107, _:L109	13	0.7
4	_:S18, _:H19, _:G20, _:N21, _:G22, _:T23, _:E24, _:K118, _:Q119, _:S122, _:V123, _:V124, _:V125, _:Q126	14	0.653
5	_:V92, _:P93, _:P94, _:V95	4	0.543
**LIFR**			
1	_:T96, _:H97, _:D98, _:L99, _:K100, _:E101, _:F125, _:E126, _:S127, _:I128, _:S129, _:G130, _:K131, _:S132, _:M152, _:H153, _:P154, _:G155, _:Q156, _:E157, _:I158, _:H159, _:N160, _:V177, _:I178, _:N179, _:V180, _:T181, _:G182, _:S183, _:Q184, _:Y185, _:I186, _:K187, _:A188, _:N189, _:S190, _:K191	38	0.763
2	_:T3, _:E4, _:T5, _:N6, _:V7, _:F8, _:C24, _:M25, _:S26, _:P27, _:T28, _:K29, _:V30, _:L31, _:S32, _:Q34, _:I35, _:G36, _:N37, _:T38, _:L39, _:R40, _:P41, _:L42, _:I43, _:H44, _:L45, _:Y46, _:G47, _:Q48, _:T49, _:V50, _:A51, _:I70, _:T71, _:D72, _:D73, _:D74, _:V75, _:Y76	40	0.731
3	_:A16, _:G17, _:S18, _:N19, _:H53, _:L55, _:N56, _:I57	8	0.585
4	_:I138, _:G140, _:L141, _:T142, _:N143, _:E144	6	0.566

Supplementary Figure 1a: full length gels of Fig 2a.

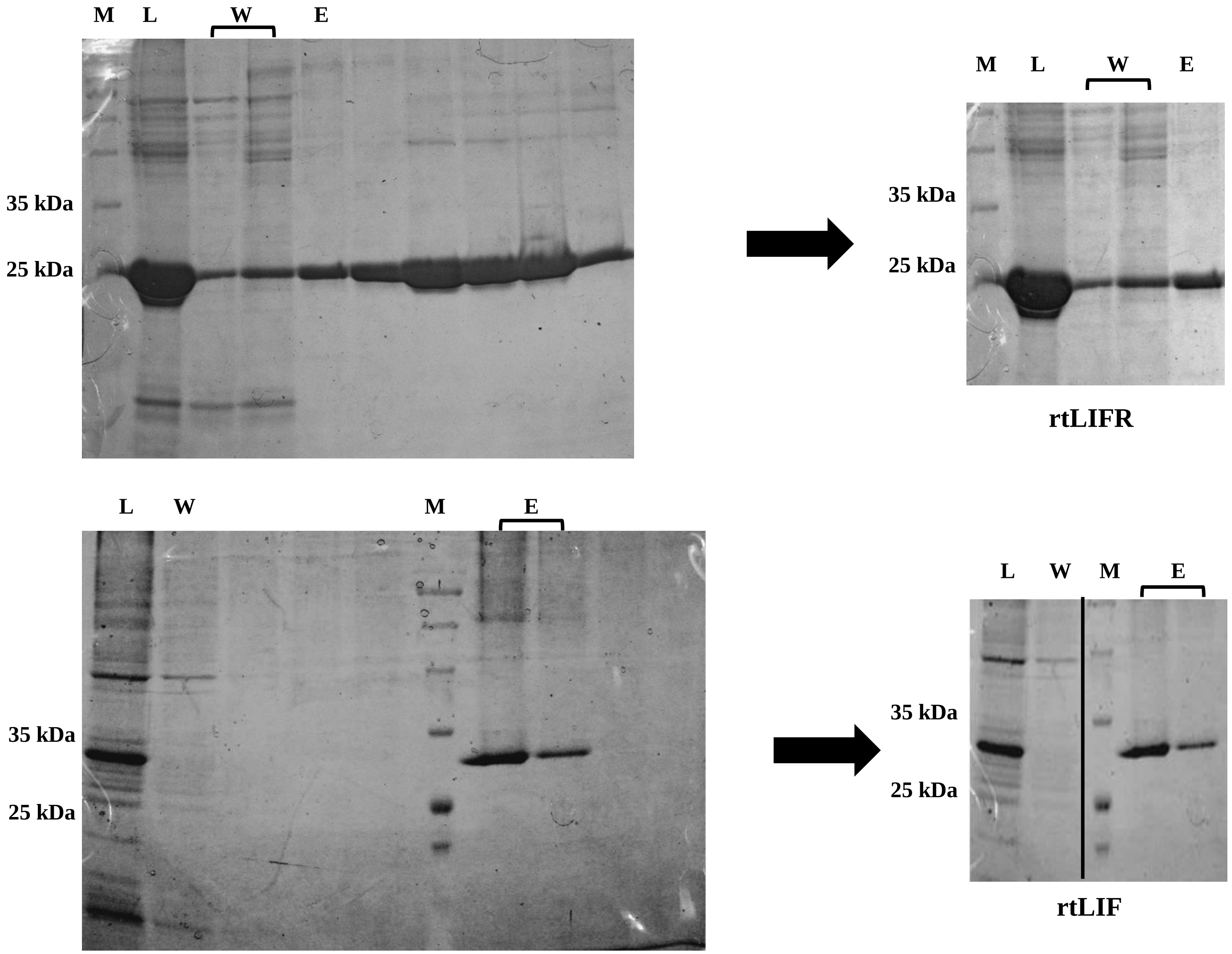

Supplementary Figure 1b: full length blot of Fig 2b.

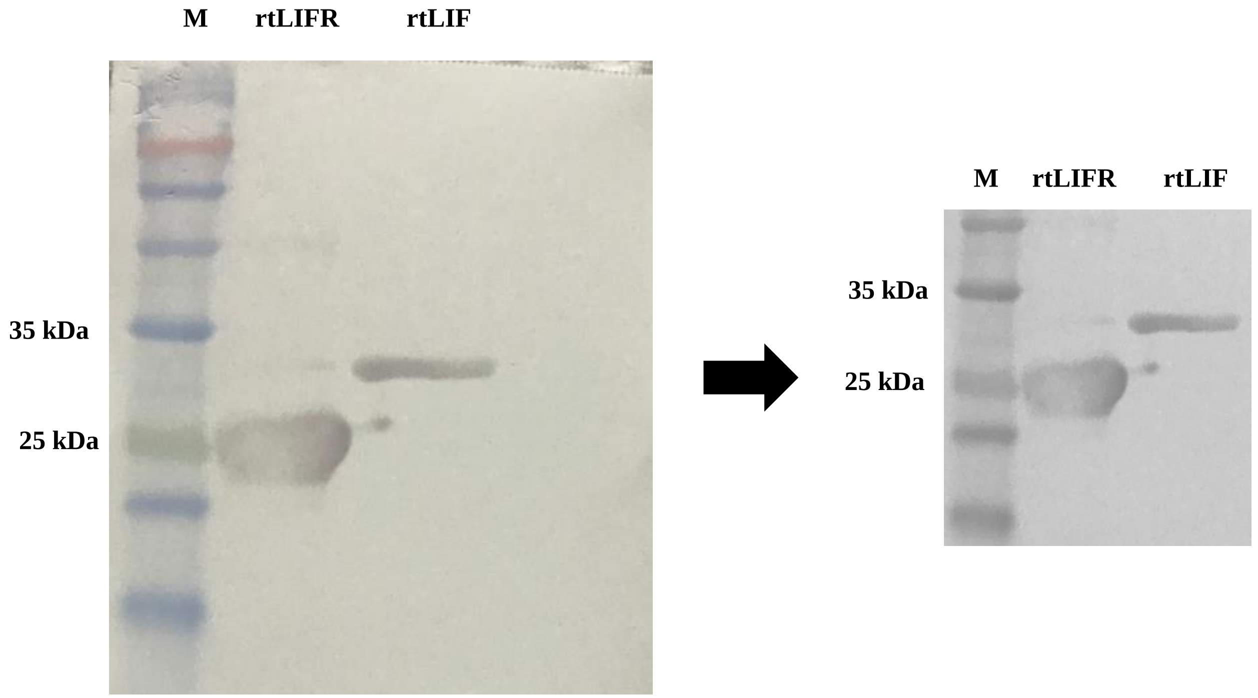

Supplement: Supplementary file 1 — Supplementary Information. [file 41598_2020_68158_MOESM1_ESM.pdf]
